# Cytoskeletal Regulation of Dermal Regeneration

**DOI:** 10.3390/cells1041313

**Published:** 2012-12-19

**Authors:** Xanthe L. Strudwick, Allison J. Cowin

**Affiliations:** 1 Wound Healing Laboratory, Women’s and Children’s Health Research Institute, 72 King William Road, North Adelaide, South Australia 5006, Australia; E-Mail: xanthe.strudwick@adelaide.edu.au; 2 Discipline of Paediatrics, University of Adelaide, South Australia 5005, Australia

**Keywords:** cytoskeleton, flightless I, gelsolin, skin regeneration, repair

## Abstract

Wound healing results in the repair of injured tissues however fibrosis and scar formation are, more often than not the unfortunate consequence of this process. The ability of lower order vertebrates and invertebrates to regenerate limbs and tissues has been all but lost in mammals; however, there are some instances where glimpses of mammalian regenerative capacity do exist. Here we describe the unlocked potential that exists in mammals that may help us understand the process of regeneration post-injury and highlight the potential role of the actin cytoskeleton in this process. The precise function and regulation of the cytoskeleton is critical to the success of the healing process and its manipulation may therefore facilitate regenerative healing. The gelsolin family of actin remodelling proteins in particular has been shown to have important functions in wound healing and family member Flightless I (Flii) is involved in both regeneration and repair. Understanding the interactions between different cytoskeletal proteins and their dynamic control of processes including cellular adhesion, contraction and motility may assist the development of therapeutics that will stimulate regeneration rather than repair.

## 1. Introduction

Tissue repair is a dynamic and complex process triggered in response to injury. When damage occurs to the skin the primary goal is to re-establish the external barrier to protect against bacterial infection and prevent fluid and temperature loss. However, the rapid reestablishment of homeostasis and barrier function is accompanied by a loss of native structures of the unwounded tissue and scar formation, with associated diminished functionality. There is therefore a great need for a more regenerative response to occur to restore functional properties and original tissue architecture [1]. While mammals have significantly limited regenerative capacity when compared to lower order vertebrates and invertebrates there are instances of regenerative strategies existing both in developing and mature tissues [2,3,4]. The actin cytoskeleton is a critical component of all cells. Cytoskeletal proteins are involved in cellular adhesion, contraction and motility all of which are fundamental to many repair processes. Understanding the relationship between the actin cytoskeleton and tissue regeneration will therefore provide insights into the repair process and potentially facilitate new approaches to improve healing.

## 2. Regeneration *vs.* Repair

**Figure 1:**
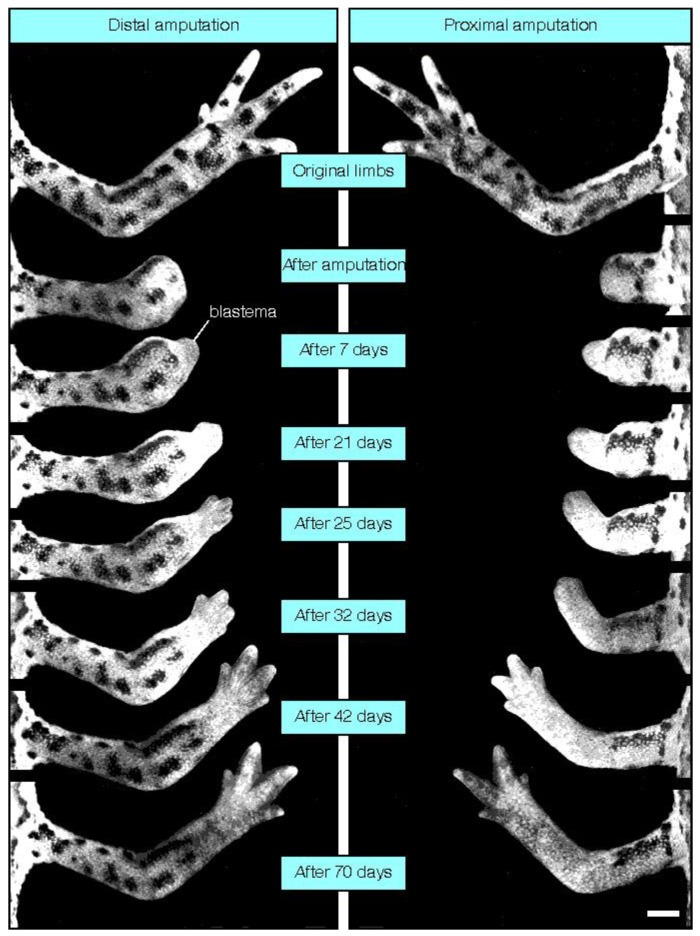
Regeneration of a salamander forelimb following distal amputation below the elbow (left) or proximal amputation through the humerus (right) (Adapted from [15]).

Regeneration of intact tissue, skin or even a limb post injury has long been the dream of surgeons and scientists alike. Lower order vertebrates and invertebrates, such as salamanders and sea stars are capable of complete limb replacement, known as epimorphic regeneration (Figure 1), however mammals have lost the capacity to regenerate tissues post injury and repair strategies are the best that can be done and more often than not result in scar formation [1]. However, the expectation exists that mammalian regeneration is a viable possibility as there are instances where regenerative strategies are observed during wound healing both in developing and mature tissues [1,2,3].

## 3. Fingertip Regeneration

**Figure 2:**
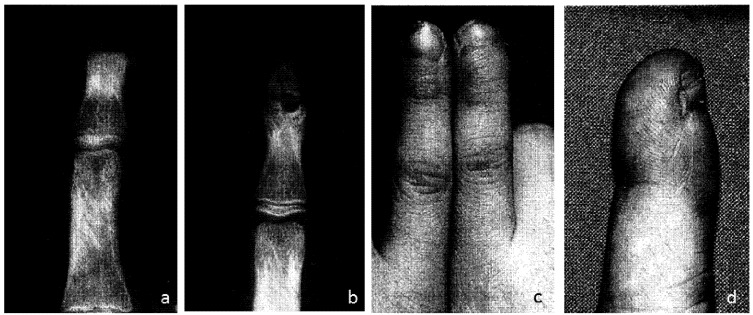
X-rays of patient at admission (**a**) and six months after the injury (**b**) showing regeneration of the distal phalanx. Photographs of the injured finger six months after the injury with left index finger for comparison (**c**) and close up (**d**) (Adapted from [16]).

Clinical reports from as early as 1932 show that humans are indeed capable of regenerating complex tissues such as the fingertip [4]. Following conservative treatment of fingertip amputations, full restoration of fingerprint, sensation and functionality has been observed with minimal visible scarring (Figure 2). Although fingertip regeneration is most commonly described in young children, adults too have demonstrated bone regrowth following conservative treatment of fingertip amputations [4,5]. Amputation resulting in the removal of the distal half of the terminal phalange results in successful regeneration of all components of tissue, including skeletal structure, with no morphological differences to the uninjured digit, although digit length may be shortened. A more proximal amputation, removing more than 2/3 of terminal phalanx, will however illicit no discernible regenerative response, although the wound will heal by cutaneous tissue repair [6,7]. 

Regeneration in the mammalian digit tip is a dynamic three phase process, incorporating reepithelialisation, dedifferentiation and redifferentiation. The first stage is marked by an initial wound healing response with inflammation, leading to the formation of an epidermal layer over the wound [8]. Although not fully investigated, it is likely this first phase closely follows the processes of cutaneous wound healing; inflammation, tissue formation and remodelling, however it is noted that regenerative wounds reepithelialise slower than non-regenerative wounds [9,10]. Wound closure time is variable in distal regenerative amputation injuries, whereas in non-regenerative proximal amputations, a single layer neo epidermis is achieved immediately following the inflammatory response, around four days post injury in mice [11]. Rates of reepithelialisation of the digit tip relate to proximal osteoclast bone degradation of the distal bone stump, resulting in the exposure of the marrow cells to the wound site and provide a route for epidermal migration [11]. It has been suggested that rapid restoration of skin integrity occurs at a cost of scar formation, with reduced structural identity and functionality [12]. The delay in reepithelialisation evident in regenerating digit tips appears to further support this hypothesis. Immediately following re-epithelialisation of the wound, dedifferentiation occurs, with the formation of a blastema under this epidermal layer contiguous with the marrow cavity centrally, and the loose connective tissue peripherally. A blastema is a devascularised, proliferating pool of progenitor cells from which organ formation or regeneration derives [7,11]. Although originally thought to be comprised of a homogenous pool of pluripotent stem cells, as is urodele regeneration, the blastema in regenerative mammalian digit tips has been found to be a heterogeneous pool of fate restricted progenitor cells [13,14]. It is during the final phase, that all tissues of the digit tip, excluding the epidermis redifferentiate from the blastema, however unlike urodele regeneration, transdifferentiation does not occur, with regenerates following fate restricted lineages [8,13].

There is a distinct difference between mammalian digit regeneration and limb regeneration in lower order vertebrates, where the same developmental techniques are employed to regrow limbs. This has lead researchers to conclude that digit regeneration seen in mammals has re-evolved from a non-regenerative condition, rather than there being an evolutionarily conserved regeneration capacity [17]. Interestingly implantation of microcarrier beads impregnated with the bone morphogenic proteins, BMP-2 and -7, under the newly formed wound epidermis of proximally amputated digits induces bone regeneration, however bone regrowth occurs via endochondral ossification, appearing to reinitiate the bone development process, rather than stimulating the regenerative response seen in distal digit amputations [10]. Investigations into digit tip regeneration using MRL mice, a strain which exhibits particularly strong regenerative capacity, have revealed the importance not only of the bone morphogenic/TGFβ pathways, critical to developmental limb formation and bone induction, as well as fracture repair, but also genes required for WNT/β-catenin mediated signalling pathways [8]. 

## 4. Mammalian Regenerative Capacity

Other potential examples of mammalian regenerative capacity do exist and include hair follicle regeneration which occurs not only as part of normal homeostasis, but also in response to wound healing. Here regeneration relies upon adult somatic stem cells providing a pool of new multipotent cells [18]. Wnt signalling is involved in hair follicle morphogenesis, and both the prolonged activation of β-catenin-dependant pathway, and forced activation of the non-canonical pathway by Wnt-5a has resulted in epidermal appendages such as rudimentary hair follicles, sebaceous glands and epithelial lined cysts forming within the healing wound area [19,20]. The human liver is one of the few internal organs which does display regenerative capacity, being able to restore lost mass following resection, from as little as 25% of its original tissue. This capacity is largely due to proliferation of and differentiation of unipotent hepatocytes [21]. Intraoral mucosal wounds also show regenerative potential, exhibiting rapid remodelling with minimal scar formation. This is due in part to a more optimal wound environment, with regards to moisture and temperature as well as decreased tension across the wound [22]. In addition though, oral mucosal fibroblasts exhibit a phenotype more similar to that seen in fetal wound healing, with enhanced MMP matrix remodelling capability resulting in less scar formation [23]. Being able to capitalise on inherent regenerative capacity of many cells types may enable regeneration of other complex organs and systems.

## 5. The Role of the Actin Cytoskeleton in Regeneration and Repair

From the initial infiltration of inflammatory cells, through the repopulation of the neodermis by fibroblasts and the reestablishment of the epidermis via keratinocyte migration, to the process of angiogenesis and tissue remodelling, effective healing of our skin and tissues relies heavily upon the dynamic reorganisation of the actin cytoskeleton [24,25]. The cytoskeleton is made up of a network of microtubules, actin filaments, intermediate filaments and their associated proteins which are responsible not only for cell shape, but also for controlling cellular adhesion, contraction and motility [25,26]. It is the active assembly and disassembly of filamentous actin and re-organisation of it into functional networks which underpins all of these important cell processes. Adhesion of cells to different surfaces and substrates is particularly required post-wounding when cellular infiltration of the wound site occurs and this is facilitated by integrin-rich focal adhesions connecting cells to underlying substrates as well as cadherin based cell-cell adhesion complexes which occur at the termination of filament bundles [27]. Not only do focal adhesions anchor polymerized actin filament stress fibres, but they facilitate the formation of actin filament bundles which provide contractile forces during cellular migration. Focal adhesion proteins, such as paxillin also serve as platforms for signal transduction which influences the extracellular matrix and facilitates dermal remodelling [28]. Thus, the precise function and regulation of the actin cytoskeleton is critical to the success of the wound healing process and its manipulation may facilitate regenerative healing.

## 6. Regenerative Fetal *vs* Reparative Adult Wound Healing

**Figure 3:**
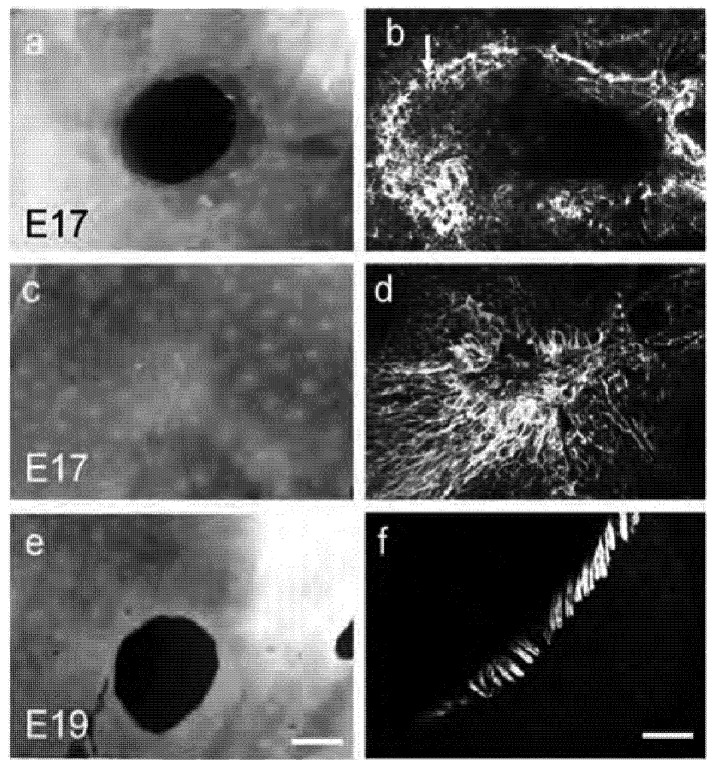
Actin cables are formed in early but not late gestation foetal skin. Wounds created in E17 but not E19 fetal skin, when cultured in DMEM/10%FBS re-epithelialise and close over 72 h. Wounded E17 fetal skin at (**a**) 3 h postwounding and (**c**) 72 h post-wounding. (**e**) wounded E19 fetal skin 72 h post-wounding. Phalloidin-FITC staining of F-actin reveals actin ring around E17 wound 48 (**b**) and 72 h post wounding (**d**). No cables are formed in E19 fetal skin wound (**f**). Magnification bar in (**e**) = 600 μm and in (**f**) = 50 μm. Arrow in (**b**) points to actin cable (Adapted from [29]).

One of the most researched areas of mammalian regeneration is that of fetal wound repair [29]. Early gestation wounds in fetal skin prior to the third trimester heal without scarring with complete restitution of dermal appendages [30,31]. Differences in fetal vs adult wound repair have been identified including reduced inflammatory responses [32,33], differences in extracellular matrix composition [34] and reduced expression of pro-scarring growth factors eg TGF-β1 [35,36]. However the actin cytoskeleton also appears to play a major role in the regenerative healing observed in the fetus [29]. Fetal wounds reepithelialise very quickly, however, rather than epidermal cells relying upon lammellipodial crawling across a provisional wound matrix the process used in adult wound repair, wound closure occurs by contraction of actin-myosin fibres in ‘purse string’ like manner, drawing the edges of the wound together (Figure 3) [9,29,37]. The purse string is formed by rapid polymerisation of filamentous actin some five to six cell back from the wound edge and appears anchored by E-cadherin-mediated adherens junctions at the leading edge to facilitate the coordinated cell movement [29,31,38]. The closure of fetal wound appears less reliant upon the mesenchymal contraction observed in adult wounds, as fetal epidermis is capable of closing a wound *in vitro* in the absence of dermal substrata [39,40]. Indeed fetal wound fibroblasts do not appear to express α-smooth muscle actin (α-SMA) suggesting fetal fibroblasts may not convert to the contractile myofibroblastic phenotype [41] indicating that they are unlikely to be responsible for wound contraction in the fetal wound [29,41]. 

In contrast to the fetus, adult wounds heal by a mechanism of repair which generally leads to scar formation and structurally and functionally diminished skin [29]. In normal skin, the collagen matrix is formed in a basket weave like pattern, allowing movement and stretch; however post injury, wound fibroblasts lay down new collagen in densely packed bundles of thinner fibres, aligned parallel to the epidermis [42]. Due to excessive elastase activity, the elastin content of this new skin is decreased within the dermis and an absence of characteristic rete ridges and normal dermal-epidermal junction can be seen [43]. The resulting scar is marked by a functional loss of elasticity, tensile strength and is prone to contracture. Skin appendages such as hair follicles and sebaceous glands never regenerate and there are clear alterations to the vascularity and nerve supply of the scar and remaining appendages. As such, scar tissue is also marked by alopecia, desiccation and thermal dysregulation [20,43]. Rapid restoration of skin integrity and protection from bacterial onslaught following injury comes with the cost of scar formation and loss of normal structure and function, which are not only unsightly but often require painful and costly revisionary surgeries [20,41]. This is particularly challenging for children post burn injury where their growth places extra demands on their skin and contracture around joints can lead to lifelong disabilities. There remains a great need for a more regenerative response to restore functional properties and original tissue architecture [20]. 

## 7. Involvement of the Actin Cytoskeleton during Regeneration and Repair

Changes in the expression of proteins associated with the actin cytoskeleton are indicative of the switch between scar-free regeneration and scar-forming repair. Paxillin colocalises with actin in E17 regenerative wounds, but not E19 reparative wounds, indicating a potential role for paxillin in wound regeneration [31]. In contrast, gelsolin is upregulated in E19 embryonic wounds but not in E17 regenerating skin and gelsolin is observed surrounding actin filaments during ‘adult type’ healing but not in the earlier, scar-free wounds [31]. Gelsolin has long been known to regulate both fetal and adult wound healing via its effect upon the actin cytoskeleton and is the founding member of a family of actin-remodelling proteins [31]. The family includes seven cytoskeletal associated proteins: gelsolin, adseverin, villin, capG, advillin, supervillin and flightless I, involved in cellular processes including motility, apoptosis and phagocytosis. All members contain a highly conserved gelsolin-like domain with either 3 or 6 homologous repeats, but may also contain additional domains endowing them with different capabilities [44]. These proteins regulate actin filaments by severing pre-existing filaments and/or capping the filament ends, a process involving the gelsolin domains. After severing, they remain attached to the ‘barbed’ ends of the broken filament, thereby preventing annealing or addition of actin monomers. Actin filaments are subsequently uncapped by interaction with phosphoinositides, leading to rapid actin assembly. This is the first step in enabling cells to reorientate their cytoskeleton to drive changes in motility, adhesion and contraction [25,45]. Gelsolin acts to scavenge actin that is exposed to extracellular spaces or released into the circulation after tissue injury preventing pathogenesis of organ injury at sites removed from the primary insult due to persistence of actin within the microvasculature [46,47].

Members of the Rho family of small GTPases; Rho, Rac and Cdc42, are largely responsible for regulating the actin cytoskeleton, coordinating the actions of multiple proteins involved in gene transcription and adhesion [27]. The assembly of stress fibres, the contractile actin-myosin filaments and associated focal adhesions are triggered by Rho activation by membrane receptors [48] enabling the stable attachment of the cell to the extracellular matrix and the transmission of force required for remodelling of the dermal matrix [49]. Rac activation is responsible for lammellipodia and membrane ruffle production at the leading edge of the motile cell through the assembly of an actin filament mesh at the cell periphery [50]. Cdc42 triggers the formation of filipodia, another actin based protrusions found at the periphery of the migrating cell [51]. Additionally, Cdc42 acts in concert with Rac to stimulate the assembly of focal complexes [51], the initial, transient adhesions formed at the periphery of the spreading or migrating cell [52]. Ras, another GTPase, also acts as a molecular switch in the regulation of cytoskeletal dynamics, as Ras activates Rac, thereby inducing lammellipodial formation [50]. Rho and Rac are also required for the formation of cadherin-based adherens junctions which link stress fibres from neighbouring cells to form tight cell-cell junctions [53]. It is clear that the dynamic control of the actin cytoskeleton underpins the processes necessary for initiation and progression of the wound healing cascade from cell infiltration of the wound site to remodelling of the dermal scar.

## 8. Flightless I in Wound Regeneration and Repair

Investigations into a more regenerative, scar free wound healing process have revealed the dynamic involvement of the gelsolin family member, Flightless I (Flii). Flii is a member of the gelsolin family of actin-remodelling proteins [54]. Flii was first identified in Drosophila melanogaster where it regulates development of flight muscles and in which Flii mutations result in irregular actin organisation and defective flight muscles [55]. The protein is highly conserved among mammals, where it is developmentally essential, with homozygous loss of Flii exhibiting gastrulation failure, leading to embryonic lethality [56]. Flii associates with many cytoskeletal proteins, colocalising with Ras and Cdc42, molecules involved in cytoskeletal reorganisation as well as localising with actin filaments themselves [57,58]. 

**Figure 4:**
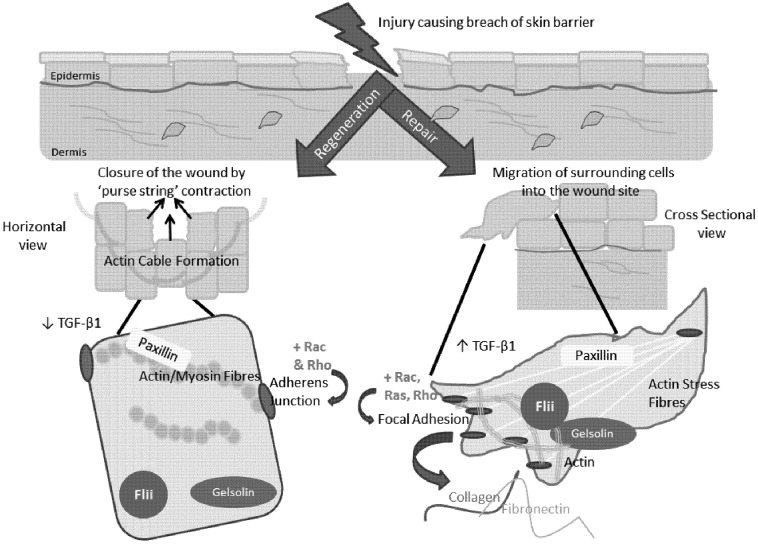
Schematic diagram of differences in actin cytoskeleton organisation and associated proteins in early gestation, regenerative healing compared to late gestation ‘adult type’ scar forming repair.

Flii is a negative regulator of wound healing, being shown to affect cell proliferation, motility and matrix production. A decrease in Flii expression, either endogenously in a heterozygous knockout engineered mouse, or via the topical application of a neutralising antibody raised against the LRR domain in murine and porcine wound models results in improved healing [12,59]. Conversely, overexpression of Flii in the mouse resulted in larger scars, with a slower, impaired wound healing response [12]. Flii localises with β-tubulin based structures involved in cell division and within lammellipodia and filopodia associated with migrating cells [60]. A decrease in Flii levels results in increased motility in keratinocytes and fibroblasts *in vitro* and in rates of keratinocyte re-epithelialisation. Decreasing *Flii* expression using siRNA in NIH 3T3 cells also results in decreased intracellular stress fiber formation [61]. A number of studies have shown a cross-talk between pathways involved in cytoskeletal remodelling, cellular adhesion and migration during wound repair, including TGF-β signaling and members of the MAP kinase family, Ras, RhoA, MAPK-ERK kinase and ERK1/2 [62,63,64,65]. Multiple *in vivo* and *in vitro* studies have demonstrated that in mouse fibroblasts, Flii specifically colocalises with cytoskeletal structures connected with a migratory phenotype [60] and associates with both structural and signaling proteins at sites of focal adhesions, including talin, paxillin and vinculin [66]. Flii is associated with actin arcs, membrane ruffles and is present at the leading edge of cells, where it also colocalizes with the GTP-binding proteins Ras, Cdc42 and RhoA that have central roles in regulating cytoskeletal reorganization [67,68]. Association of Flii LRR domain with Ras and Cdc42 proteins suggests possible involvement of Flii in downstream PI3K and MAPK signalling pathways. The PI3K/Akt signalling pathways are known to regulate numerous fundamental cellular functions during wound repair, including cell growth, proliferation, motility and survival and is activated by a variety of extracellular signals [69]. That Flii is endogenously upregulated in response to wounding and yet overexpression leads to impaired wound healing is a quirk similar to that of TGFβ1 in which expression in also upregulated in response to wounding but overexpression leads to increased scar formation [12,70] and indicates the tightly regulated nature of molecules critical to wound healing processes. Indeed in fetal skin, Flii expression in mice increases between embryonic day E17 at which the fetus heals without scar and embryonic day E19, resulting in scar formation [58]. Although wounding transiently increases Flii expression in E17 but not E19 wounds, Flii expression is down regulated in E17 keratinocytes immediately adjacent the wound. Moreover, in E19 wounds, Flii is strongly expressed within the cytoplasm and nucleus of keratinocytes at the leading edge [58]. It appears that an increase in Flii expression is linked with impaired wound healing and a switch to reparative, scar forming wound healing (Figure 4). Using a recognized model of hair follicle regeneration, recent studies by Waters *et al.*, have shown that Flii has a positive influence on hair follicle regeneration, which is contrary to its negative influences on wound healing [71]. Regenerated follicles expressing high levels of *Flii*, produced significantly longer terminal hair fibers while *Flii* deficiency resulted in a delayed or impaired regenerative potential of hair follicles [71]. *Flii^+/−^* follicles that failed to regenerate the endbulb structures also displayed low expression of markers of normal hair follicle development suggesting a lack in developmental activity [71]. The effect of Flii on cellular adhesion and its involvement in signalling pathways important to hair follicle development, growth and cycling might explain the delayed regeneration of hair follicles in *Flii* deficient mice [71]. Indeed, Flii has been shown to inhibit the Wnt/β-catenin pathway [72] and play a role as a thyroid hormone and estrogen-activated nuclear receptor co-activator [73,74] both of which would have particular significance to hair follicle development. 

Flii not only contains six repeats of the gelsolin-like domain, endowing it with actin binding abilities, but contains the novel feature among gelsolin family members of an amino-terminal leucine rich repeat sequence [57]. Leucine rich repeat motifs are known to mediate protein-protein interactions and it has been suggested this domain enables Flii to link signal transduction to cytoskeletal regulation [54,75]. Flii has both cytoplasmic and nuclear activity where it is a transcriptional coactivator. It is known to regulate transcription of the estrogen and thyroid receptors [74] and has a differential effect upon cell cycle progression [76]. Flii is secreted into human plasma and has immunological roles further indicating the critical role for Flii in wound healing [77]. Flii interacts with CARM1 and GRIP1 which are positive transcriptional regulators of the β-catenin: TCF/LEF complex within the canonical Wnt signalling pathway to inhibit β-catenin dependant transcription [72,74,78] Interestingly the cytoplasmic protein known as the murine Flightless Associated Protein 1 (FLAP1) or its human homologue Leucine Rich Repeat In FLII Interacting Protein 1/2 (LRRFIP1/2) activate this transcription pathway. FLAP1 increases IFN-β expression by enhancing β-catenin activation [79]. LRRFIP binds Dishevelled complexed with GSK-3β, which prevents GSK-3β phosphorylation of β-catenin, subsequently leading to increased transcription of downstream β-catenin targets [80]. It appears that Flii exerts its action upon the canonical WNT pathway by disrupting the binding of FLAP1/LRRFIP1/2 and β-catenin [72].

Both Flii expression and Wnt signalling is closely linked to the switch from fetal scarless to scarring healing. Wnt signalling is induced at embryonic day 19.5 in mice, but not before, whilst Flii expression is increased significantly at embryonic day 19 [58,81]. The involvement of Flii and the Wnt signalling pathways in the switch between fetal scar free healing and wound repair, as well as hair follicle regeneration combined with the dynamic interactions between Flii and both the canonical and noncanonical signalling pathways, suggests a potential role in regeneration as well as a possible target for therapeutic manipulation leading to enhance wound healing outcomes.

## 9. Conclusion

Although mammals have limited regenerative capacity when compared to the epimorphic regeneration seen in lower order vertebrates and invertebrates, there remains instances in which regenerative healing can occur, returned full structure and function to the injured tissue. As well as select tissues displaying this capacity, such as the liver, oral mucosa, hair follicles and the tissues of the fingertip, fetal wounds heal with scar free restoration, due in a large part, to differential organization of the cellular cytoskeleton. This network of actin filaments and microtubules which give the cell its structure are dynamically regulated to facilitate adhesion, motility and contraction as well as division, processes fundamental to all processes of wound healing. Differences in expression levels and localization of cytoskeletal proteins of the gelsolin family between regenerative healing, such as in fetal skin, and the more common adult type, scar forming healing process, has highlighted the importance of cytoskeletal regulation in tissue regeneration. The dynamic involvement of family member, Flii appears critical to the regulation of wound healing and is a key player in the drive towards regenerative healing. Further insights into the events important for tissue regeneration will identify further targets for therapeutic development. To be able to drive the body towards the replacement of native tissues, rather than merely repairing the injury offers great potential to improve the quality of life of humans worldwide. Understanding the differences between regeneration and repair offers the greatest prospect that we will one day be able to stimulate this regenerative process and identifying the role of the actin cytoskeleton in this process is likely to be a critical step towards this goal.
